# Precipitating factors and clinical outcomes of diabetic ketoacidosis in Eastern Cape, South Africa

**DOI:** 10.4102/jcmsa.v3i1.99

**Published:** 2025-02-17

**Authors:** Siqhamo Magadla, Chukwuma Ekpebegh, Nomagugu Ndlovu, Thozama Dubula, Sibi Joseph

**Affiliations:** 1 Department of Internal Medicine, Faculty of Health Sciences, Walter Sisulu University, Mthatha, South Africa; 2 Department of Biology and Environmental Sciences, Faculty of Natural Sciences, Walter Sisulu University, Mthatha, South Africa; 3 Department of Internal Medicine and Pharmacology, Faculty of Medicine and Health Sciences, Walter Sisulu University, Mthatha, South Africa

**Keywords:** diabetic ketoacidosis, clinical features, outcomes, precipitating factors, mortality

## Abstract

**Background:**

Diabetic ketoacidosis (DKA) is a common hyperglycaemic emergency in persons living with diabetes (PLWD), and outcomes of treatment depend on the precipitating factor.

**Methods:**

A cross-sectional prospective study of patients admitted in the adult high care unit of Nelson Mandela Central Hospital was carried out from 19 February 2022 to 19 January 2023. Patients were assessed for demographic, clinical profiles and outcomes concerning precipitating factors. The outcomes were duration of admission and discharge from the hospital as alive or dead.

**Results:**

There were 55 PLWD, all black African, predominantly females, known with diabetes with a mean age of 38 ± 15.8 years. The main precipitants for DKA in descending order were infections (47%), treatment omission (30%) and a new diagnosis of diabetes (19%). The mean duration of admission for all patients was 8.2 ± 5.3 days. The length of hospital stay was 9.5 ± 5.2 days, 5.6 ± 2.2 days, and 8.6 ± 7.6 days, respectively (*p* = 0.118). The mortality in all patients was 9 (16.4%), and all but one death was associated with sepsis. There were no significant differences in the HbA1c among patients with infection (13.9 ± 3.8), those who omitted treatment (12.6 ± 4.7) and those newly diagnosed (12.2 ± 2.2) (*p* = 0.620).

**Conclusion:**

The high mortality rates in our DKA patients were mainly related to infections. The high HbA1c indicates poor glycaemic control preceding DKA. Improving glycaemic control, preventing infections, and early treatment of infections can reduce DKA-related mortality among patients.

**Contribution:**

This study provides a comprehensive analysis of DKA in resource-limited settings, focusing on its precipitating factors, clinical profiles, and outcomes among adults in the Eastern Cape, South Africa. Infections were identified as the leading precipitant, with poor glycaemic control prevalent across all cases and a mortality rate of 16.4%, primarily due to sepsis. The findings highlight the urgent need for strategies to improve glycaemic control, prevent infections, and ensure timely interventions to reduce DKA-related mortality. This research aligns with the *Journal of the Colleges of Medicine of South Africa*’s mission to advance clinical practice by addressing critical healthcare challenges in underserved communities, offering insights applicable across the region.

## Introduction

Diabetes is a major public health challenge with an increasing prevalence worldwide.^1^ The number of adults living with diabetes worldwide increased from 108 million in 1980 to 422 million in 2014.^1^ The International Diabetes Federation (IDF) has predicted that the number of persons living with diabetes worldwide will increase by 50% in 2030 from 366 million in 2011.^2^ Mainly around two-thirds of diabetic ketoacidosis (DKA) cases occur in a patient with type 1 diabetes mellitus (DM) and one-third occur in type 2 DM patients.^3^ Complications of diabetes are common among patients with type 1 or type 2 DM and are responsible for significant morbidity and mortality.^4^ Diabetic ketoacidosis is one of the most common acute hyperglycaemic emergency in persons living with diabetes.^5^ It is characterised by hyperglycaemia, ketosis and high anion gap metabolic acidosis. Diabetic ketoacidosis can also occur in women with gestational diabetes and patients with type 2 DM. Since the early 2000s, the global incidence of DKA has increased.^6^ This condition represents a severe, life-threatening complication of diabetes that may result in diabetic coma or death.^7^ The cause of death in patients with DKA relates to the underlying precipitating cause and advanced age.^5^ Infection is the most frequent precipitant worldwide accounting for 30% – 50% of cases.^8^ Treatment non-compliance is also a significant precipitating factor.^9^ The other precipitating factors include a new diagnosis of diabetes, myocardial infarction, cerebrovascular accident, surgery, pregnancy, pancreatitis, steroid use and psychosocial factors such as eating disorders and substance abuse.^10,11^

In this cross-sectional study, we report on clinical profiles and outcomes following admissions for DKA in association with various precipitating factors.

## Research methods and design

### Study design

Cross-sectional study on adults admitted with DKA at Nelson Mandela Central Hospital (NMCH), Eastern Cape province, South Africa.

### Study setting

The study was conducted at NMCH, Mthatha, Eastern Cape province, South Africa, from 19 February 2022 to 19 January 2023. The NMCH has a catchment area with approximate population of 1.5 million people residing in peri-urban and rural areas.

### Inclusion criteria

Age ≥ 13 years with DKA.

### Exclusion criteria

Age < 13 years, hyperglycaemic emergencies not meeting the criteria for DKA and refusal to grant consent.

### Severity of diabetic ketoacidosis

Severity of DKA was classified as shown in Table 1.^12^

**TABLE 1:** Classification of diabetic ketoacidosis severity.

Variable	Mild	Moderate	Severe
Arterial pH	7.25–7.30	7.00 – ˂ 7.24	˂ 7.00
Serum bicarbonate	15–18 mEq/L	10 – ˂ 15 mEq/L	˂ 10 mEq/L
Serum ketone	Positive	Positive	Positive
Urine ketone	Positive	Positive	Positive
Effective serum osmolality	Variable	Variable	Variable
Anion gap	˃ 10 mEql/L	˃ 12 mEq/L	˃ 12 mEq/L
Mental status	Alert	Alert/drowsy	Stupor/coma
Plasma glucose	˃ 13.88 mmol/L	˃ 13.88 mmol/L	˃ 13.88 mmol/L

*Source*: Adapted from American Diabetes Association. Standards of medical care in diabetes – 2009. Diabetes Care. 2009;32(Suppl. 1):S13–S61. https://doi.org/10.2337/dc09-S013

### Data collection

A data collection sheet was used to collect demographic information (age, gender, race), diabetes type (classified based on features which include family history of diabetes, increased body mass index [BMI] and presence of acanthosis nigricans), duration of diabetes, baseline co-morbidities, duration of DKA symptoms, precipitating factors, laboratory parameters and vital signs (pulse, blood pressure, level of consciousness reflected as alert, drowsy, confused, stuporous and comatose using alert, voice, pain, unresponsiveness [AVPU] scale) at presentation, DKA severity, duration of admission, discharge state as dead or alive. Laboratory results were retrieved from National Health Laboratory Service (NHLS) Track Care system. All data were transcribed to a Microsoft^®^ Excel spread sheet.

### Data analysis

Data were analysed using Statistical Package for Social Science (SPSS version 24, Chicago, Illinois, United States [US]). Categorical variables are reported as proportions and compared using Chi-Square test. Continuous variables are depicted as mean and standard deviation if normally distributed or median and interquartile range if not normally distributed. The level of significance was set at *p* ˂ 0.05.

### Ethical considerations

Ethical approval was obtained from the Research and Ethics Committee, Faculty of Health Sciences, Walter Sisulu University with research protocol number 118/2021. Permission to conduct the study was also obtained from Eastern Cape Department of Health through the Nelson Mandela Central Hospital (NMCH) management (approved EC_202201_031). Written informed consent was obtained from participants or their relatives where patient was unable to give consent. All participants less than 18 years of age granted assent in addition to consent from parents or guardians. For patients with altered sensorium, consent and assent were sought for when fully conscious.

## Results

In total, 55 participants with DKA were included, all black Africans with a mean age of 38 ± 15.8 years and range of 13–70 years. The proportion of females to males was 3:1. Most participants (80%) were known to have diabetes, while 20% were newly diagnosed with DM at this presentation for DKA. Most participants (43.6%) had severe DKA, followed by 33.4% with moderate DKA and 20% with mild DKA. Diabetes was mainly type 2 regardless of DKA severity as shown in Table 2. The mean age of those omitting treatment was 29.6 ± 13.8, years lower than those with infection, 42.3 ± 14.9 or new diagnosis of DM, 36.9 ± 17.4 (*p* = 0.03).

**TABLE 2:** Clinico-demographic profile of study participants according to precipitants.

Variable	Infection	Treatment omission	New diagnosis of diabetes	*p*
Age (years)	42.3 ± 14.9	29.6 ± 13.8	36.9 ± 17.4	0.030
Females (%)	73.1	63	80	0.530
**Diabetes type**				
Type 1	11	8	15	0.001
Type 2	16	27	30	0.077
**Number of patients with various DKA severities**				
Mild	1	4	4	0.110
Moderate	11	6	2	0.665
Severe	14	6	4	0.235
**Number of patients with various comorbidities**				
Hypertension	5	2	2	0.369
HIV	7	1	2	0.206

DKA, diabetic ketoacidosis; HIV, human immunodeficiency virus.

The majority of patients (61.8%, *n* = 34/55) had an altered sensorium at presentation. Only one patient had glycosylated haemoglobin (HbA1c) below 7%.

Table 3 shows that C-reactive protein (CRP) was significantly related to the precipitating factors (*p* = 0.001), with higher CRP following infection than a new diagnosis of diabetes and treatment omission.

**TABLE 3:** Clinico-laboratory parameters of study participants according to precipitants.

Variable	Infection (*n* = 26)	%	Treatment omission (*n* = 16)	%	Newly diagnosed (*n* = 10)	%	*p*
**Level of consciousness**							
*Unconscious (%)	54	14	75	12	50	5	0.500
SBP < 90 mmHg (%)	85	22	100	16	80	8	0.290
HbA1c	13.9 ± 3.8	-	12.6 ± 4.7	-	12.2 ± 2.2	-	0.620
CRP (mg/L)	162.3 ± 117.9	-	19.69 ± 27.2	-	3.9 ± 24.1	-	0.001

SBP, systolic blood pressure; HbA1c, glycated haemoglobin; CRP, C-reactive protein.

**FIGURE 1:**
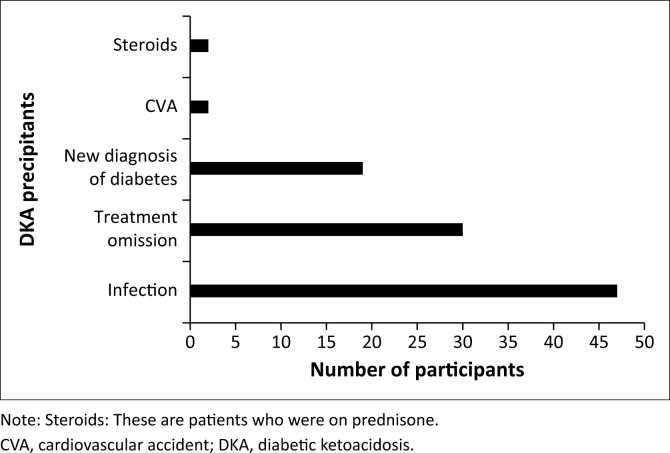
Diabetic ketoacidosis precipitants among study participants (%).

Figure 1 shows that infections, treatment omission and a new diagnosis of DM were the main precipitants of DKA.

The mean duration of hospital stay for all 55 participants was 8.2 ± 5.3 days, and a mortality of 16.4% (*n* = 9/55) was noted. The duration of admission for infection, treatment omission and a new diagnosis of diabetes are 9.5 ± 5.2, 5.6 ± 2.2 and 8.6 ± 7.6 days, respectively (*p* = 0.118). Eight of nine patients who demised had infection as precipitating cause of DKA, while the ninth person who demised had a cerebrovascular accident.

Table 4 shows that patients who died were middle-aged to elderly and all had type 2 DM.

**TABLE 4:** Clinico demographic profiles of participants who demised.

Pt (*n* = 9)	Age (years)	Gender	DM type	DKA severity	Comorbidities	Precipitant	HbA1c (%)	LOHS (days)
1	64	M	2	Moderate	HPT	Infection	12.0	4
2	57	F	2	Moderate	HPT and HIV	Infection	12.8	5
3	54	F	2	Moderate	HIV	Infection	12.5	14
4	39	F	2	Severe	HPT	Infection	10.0	13
5	41	F	2	Severe	None	Infection	15.0	3
6	50	F	2	Moderate	HPT and HIV	CVA	10.7	4
7	57	M	2	Mild	HPT	Infection	17.7	10
8	66	M	2	Moderate	None	Infection	8.5	8
9	45	M	2	Moderate	None	Infection	10.8	4

M, male; F, female; DM, diabetes mellitus; DKA, diabetic ketoacidosis; HIV, human immunodeficiency virus; HPT, hypertension; HbA1c, glycated haemoglobin; CVA, cardiovascular accident; LOHS, length of hospital stay; Pt, patient.

Table 5 shows that foot gangrene was the most prevalent infection.

**TABLE 5:** Site of infections among demised participants.

Infection	Number of participants	Percentage of participants (%)
Chest infection	2	22.2
Foot gangrene	4	44.4
Perianal abscess	1	11.1
Necrotising fasciitis breast	1	11.1
None	1	11.1

Note: None: The patient demised, however, with no documented infection.

## Discussion

A cross-sectional prospective study was conducted on 55 patients admitted to the adult high-care unit at NMCH between February 2022 and January 2023. These patients were examined for their demographic characteristics, clinical profiles and treatment outcomes based on the factors triggering DKA.

The finding of female preponderance among patients presenting with DKA is likely a reflection of the demographics of the background population. In addition, this could also mean that females are better at health-seeking behaviour.

Hypotension (identified by SBP < 90, shown in Table 2) and altered sensorium at presentation may be related to such factors as dehydration from fluid loss and sepsis. These clinical features in most patients may indicate late presentation to hospital.

The study shows infections, treatment omission and a new diagnosis of diabetes as the main precipitants of DKA at 47%, 30% and 19%, respectively. The higher treatment omission rate observed in younger patients is likely related to poor compliance with multiple insulin injections, as 9 of 16 patients with treatment omission were type 1 DM. The infections in our patients included severe infections such as wet gangrene of the lower limbs and necrotising fasciitis. Our findings of infections as the major precipitant of DKA are similar to those from indigent and rural settings resembling ours.^7,13^ They, however, differ from studies conducted in Saudi Arabia and Israel, which found treatment omission as the main precipitant.^14,15^ This underscores the need for prompt identification and treatment of infections in persons with diabetes by all healthcare practitioners involved in diabetes management. Although infection is a known precipitant for DKA, as reported in other studies,^16,17^ poor glycaemic index is a trigger for DKA given that in our study all the 3 groups had poor glycaemic control as shown by high HBA1c levels. Very poor glycaemic control was observed in both known diabetes and newly diagnosed patients.

Although DKA was long considered a key clinical feature of type 1 DM but, in recent years, an increasing number of ketoacidosis cases have also been reported in subjects with type 2 DM.^18^ In the United States (US) 34% of DKA episodes occur in patients with type 2 DM.^19^ American and European retrospective studies show that approximately 20% – 30% of DKA patients had type 2 DM.^20,21^ A study carried out in Italy recently reported that trends of DKA in adult patients with type 2 DM have been increasing in both male and female.^22^

The finding that 19% of patients were newly diagnosed with diabetes at index presentation with DKA is likely a reflection of the increasing prevalence of diabetes in our population. There were 10 patients newly diagnosed with diabetes of who eight were classified as type 2 DM. The HbA1c levels of 10.1% – 16.4% in these patients just diagnosed with diabetes at DKA presentation is in keeping with a pre-existing chronic rather than acute hyperglycaemia. This underscores the need for screening programmes for persons at risk of diabetes and thus of DKA such as the overweight and obese, hypertensive and previous gestational diabetes.

The mortality rate of 16.36% compared with that of other centres in African countries^23,24^ but was higher than the 5.00% rate reported from the United Kingdom (UK) and US.^21,22^ Patients from well-resourced centres are less likely to present with severe infections as they are more likely to receive prompt treatment. Remarkably, no patient with treatment omission or a new diagnosis of diabetes died. The low mortality rates among patients experiencing treatment omission may be attributed to the prompt correction of metabolic derangements, which were readily reversible with intravenous fluids and insulin therapy. This is in contrast with patients with an infective precipitant in whom appropriate antimicrobial therapy, source control, among others, is required to mitigate morality. Furthermore, patients with an infection are more likely to be sick with more deranged parameters requiring correction. Conversely, the treatment of infections typically depends on identifying the causative organism and administering appropriate antimicrobials in correct dosages for an adequate duration. In some cases, escalation to surgical interventions, such as wound debridement and amputations, may be necessary to prevent mortality.

### Limitations

Our classification of diabetes type was on clinical grounds, as we did not do auto-antibodies such as anti-glutamic acid decarboxylase antibody and anti-insulin antibody to diagnose type 1 DM.

## Conclusion

Our DKA patients had very poor glycaemic control and were hypotensive with altered sensorium at presentation. The majority of patients had type 2 DM. Infections, treatment omission and a new diagnosis of diabetes were the main precipitating factors. Deaths were mainly in patients with infections as the precipitating factor. The poor glycaemic control observed in this study underscores the importance of improved glycaemic control through educative programmes and the need for early screening for patients with risk factors for diabetes. Further studies may need to be undertaken to understand the factors underlying poor glycaemic control.
